# Severely deranged vital signs as triggers for acute treatment modifications on an intensive care unit in a low-income country

**DOI:** 10.1186/s13104-015-1275-9

**Published:** 2015-07-25

**Authors:** Carl Otto Schell, Markus Castegren, Edwin Lugazia, Jonas Blixt, Moses Mulungu, David Konrad, Tim Baker

**Affiliations:** 1 Centre for Clinical Research Sörmland, Uppsala University, Uppsala, Sweden; 2 Department of Internal Medicine, Medicinkliniken, Nyköping Hospital, Sörmland County Council, 61185 Nyköping, Sweden; 3 Department of Anaesthesia and Intensive Care, Muhimbili University of Health and Allied Sciences, Dar es Salaam, Tanzania; 4 Department of Anaesthesia, Intensive Care and Surgical Services, Karolinska University Hospital, Stockholm, Sweden; 5 Department of Physiology and Pharmacology, Karolinska Institutet, Stockholm, Sweden; 6 Department of Anaesthesia and Intensive Care, Muhimbili National Hospital, Dar es Salaam, Tanzania; 7 Department of Physiology and Pharmacology, Karolinska Institutet, Stockholm, Sweden; 8 Global Health - Health Systems and Policy, Department of Public Health Sciences, Karolinska Institutet, Stockholm, Sweden

**Keywords:** Critical care, Vital signs, Task shifting, Intensive Care Unit, Goal directed therapy, Early warning scores, Quality of care, Low income countries, Developing countries, Tanzania

## Abstract

**Background:**

Critical care saves lives of the young with reversible disease. Little is known about critical care services in low-income countries. In a setting with a shortage of doctors the actions of the nurse bedside are likely to have a major impact on the outcome of critically ill patients with rapidly changing physiology. Identification of severely deranged vital signs and subsequent treatment modifications are the basis of modern routines in critical care, for example goal directed therapy and rapid response teams. This study assesses how often severely deranged vital signs trigger an acute treatment modification on an Intensive Care Unit (ICU) in Tanzania.

**Methods:**

A medical records based, observational study. Vital signs (conscious level, respiratory rate, oxygen saturation, heart rate and systolic blood pressure) were collected as repeated point prevalences three times per day in a 1-month period for all adult patients on the ICU. Severely deranged vital signs were identified and treatment modifications within 1 h were noted.

**Results:**

Of 615 vital signs studied, 126 (18%) were severely deranged. An acute treatment modification was in total indicated in 53 situations and was carried out three times (6%) (2/32 for hypotension, 0/8 for tachypnoea, 1/6 for tachycardia, 0/4 for unconsciousness and 0/3 for hypoxia).

**Conclusions:**

This study suggests that severely deranged vital signs are common and infrequently lead to acute treatment modifications on an ICU in a low-income country. There may be potential to improve outcome if nurses are guided to administer acute treatment modifications by using a vital sign directed approach. A prospective study of a vital sign directed therapy protocol is underway.

## Background

Global critical care capacity is increasing [1–3]. While low income countries (LIC) have the greatest burden of critical illness, little is known about the state of their critical care services [2, 4–6]. Critical care has previously not been considered a cost-effective part of the health systems of LIC [7]. This may have been due to a perception that critical care invariably involves expensive treatments and equipment, such as in Intensive Care Units (ICUs) in high-income countries (HIC). However, evidence is growing that several aspects of critical care, such as close monitoring by nurses, maintaining a free airway, oxygen therapy, intravenous fluid resuscitation and some acute surgical operations may be cost-effective in LIC, even when compared to preventive care approaches [8–10]. In its potential to save lives of the young and previously healthy with reversible disease, it deserves increased attention from researchers and policy-makers [2, 4, 11, 12].

A fundament of critical care is the recognition of severely deranged physiology and subsequent acute treatment modifications. The patients’ vital signs give useful information about physiological derangement and have been shown to correlate with negative outcomes such as cardiac arrest and death [13–16]. The use of vital signs as triggers for acute treatment modifications is the basis of modern routines in critical care, for example goal directed therapy [17] and rapid response teams [18, 19]. It is not known to what extent ICUs in LICs utilise goal directed therapies or severely deranged vital signs as treatment triggers.

Tanzania is a LIC in East Africa with a population of 44 million. Health expenditure in Tanzania is 27 USD per person per year [20]. Critical Care is in its infancy in Tanzania. Most hospitals do not have an ICU, and of the 820 doctors in Tanzania, only 15 are specialists in critical care [21] (personal communication: Dr U Mpoki, president of the Society of Anaesthesiologists of Tanzania).

This study, the first from the interventional vital signs directed therapy (VSDT) research collaboration, aims to assess how often severely deranged vital signs trigger an acute treatment modification on an ICU in Tanzania.

## Methods

An observational, medical records based study from the 6-bedded ICU in Muhimbili National Hospital (MNH) in Dar es Salaam, Tanzania.

### Ethics

Ethical clearance was granted by the National Institute of Medical Research in Tanzania (NIMR/HQ/R.8a/Vol.IX/1606), Muhimbili University of Health and Allied Sciences (MU/DRP/AEC/Vol.XVI/125) and the Ethical Review Board in Stockholm (EPN/2015/673-31/2). Permission for the study was granted by The Tanzanian Commission for Science and Technology and by MNH. As the study was part of quality improvement on the ICU, individual patient consent was not required by the ethical committees.

### Setting

Muhimbili National Hospital is a national referral hospital with 900 beds. During the study period 5 anaesthesiologists shared the workload in the hospital, including 24 h medical cover for the 11 operating theatres and the ICU. There were 31 nurses employed on the ICU, of which one had a formal critical care education. There were 4–6 nurses working per shift. All ICU-beds were equipped with respirators and non-invasive monitoring of vital signs. Available par-enteral treatment included crystalloids, blood transfusion, antibiotics and the vasoactive drug Dopamine. The laboratory services took approximately 6 h for standard analyses including arterial blood gases, blood counts and chemistry. Standard X-rays were available but the hospital CT scanner was not functioning. The study was conducted as a part of the Muhimbili–Karolinska Anaesthesia and Intensive Care Collaboration (MKAIC), an international partnership that has been working to investigate and improve care at Muhimbili since 2008.

### Data

**Figure 1:**
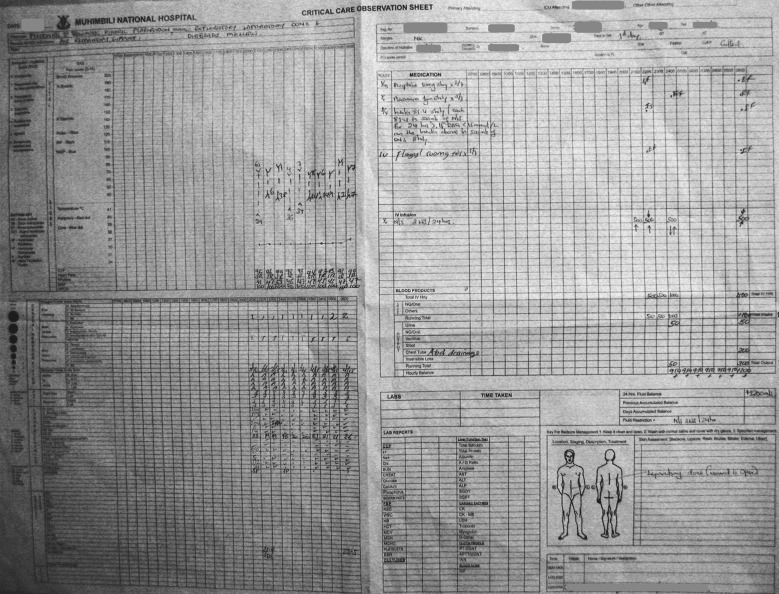
Data source: handwritten observation charts from the ICU. The ICU observation charts contain nurses’ records of vital signs, patients’ position, treatments administered and fluid balances every hour. There is also information on diagnosis, other medical treatments and care given.

All patients over 16 years admitted to the ICU in a 1 month period were identified and data from that month were included. The patients were identified from the ICU-admission book and their medical notes were retrieved. Handwritten ICU Observation Charts were found within the notes. The ICU Observation Charts contain nurses’ records of vital signs, treatments administered and fluid balances every hour (Figure 1).

Five vital signs were studied: conscious level, respiratory rate, oxygen saturation, heart rate and systolic blood pressure. We extracted the vital signs observations as repeated point prevalences (observation-timepoints) every 8 h (08:00, 16:00, 24:00). This interval was chosen as a trade-off to capture most changes in patient condition but avoiding dependency on the most severely ill patients. For some patients, documentation by the nurses had been reduced to 2- or 3-h and in the case of missing data for that reason; values between 1 h earlier and 2 h later were used.

When a severely deranged vital sign was identified we noted whether any treatment modification was carried out within the following hour and whether this treatment modification was appropriate.

### The vital signs directed therapy protocol

**Figure 2:**
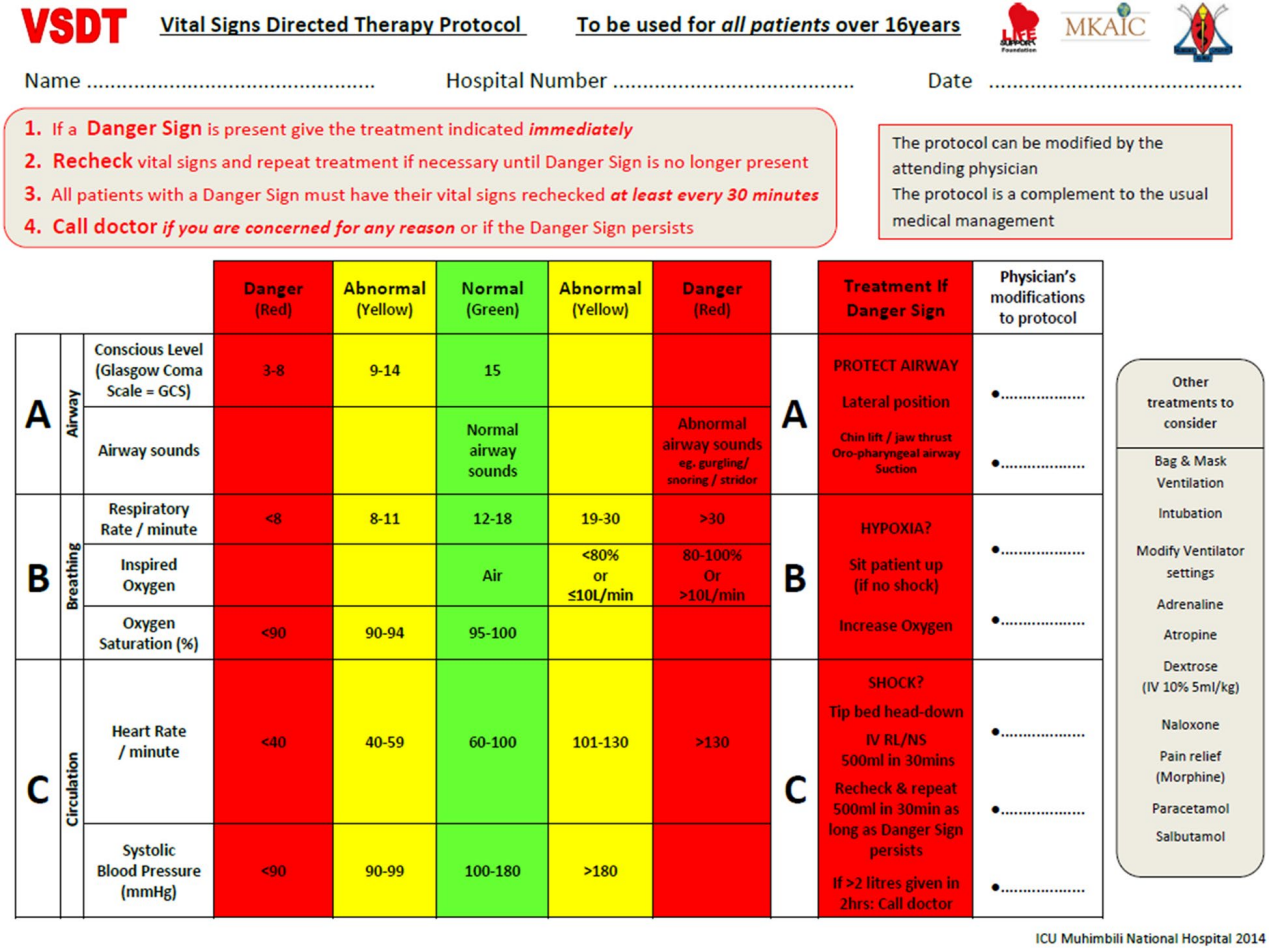
The vital signs directed therapy (VSDT) protocol. Vital sign observations are categorized as normal (*green*), abnormal (*yellow*) and danger signs (*red*). A danger sign calls for immediate action by the nurse.

Severely deranged vital signs and appropriate acute treatment modifications were defined from the vital signs directed therapy (VSDT) protocol (Figure 2). The VSDT protocol is the result of a 2 year international collaborative process between the authors of this study and other experts in the MKAIC collaboration and the European Society of Intensive Care Medicine (ESICM) group for Global Intensive Care.

VSDT utilises seven vital signs. Vital sign observations are categorized as normal (green), abnormal (yellow) and danger signs (red). The cut-offs for danger signs are based on the Karolinska Hospital Medical Emergency Team (MET) triggers [22] and the ESICM Vital Nursing Card (Unpublished). A danger sign calls for immediate treatment by the nurse. Every vital sign extracted for the study was assigned the corresponding VSDT-colour. At the time of this study, the VSDT protocol was not used in clinical practice.

### Data analysis

SPSS (IBM^®^) was used for descriptive statistics. Due to sample size, statistical inferences were not made. Numerical data are presented as median and interquartile range.

## Results

15 adult patients were admitted to the ICU during the study period. Nine were female and the median age was 37 years (28–46). Four patients died in the ICU (25%). Diagnoses are shown in Table 1. Out of 15 patients, 13 medical records were found in the archives. ICU observation charts were found from 9 patients and vital signs data from 126 observation-timepoints. For the patients with ICU observation charts available, a total of nine charts were missing from the notes.

**Table 1:** Study patients

Patient	Diagnosis	Days in ICU	Died in ICU	Medical record available	Observation charts available	No. of observation-timepoints from patient
1	Kidney stone, post-op nephrostomy	4	Yes	Yes	Yes	4
2	Peritonitis, post-op (2 operations)	7	Yes	Yes	Yes	51
3	Pulmonary oedema, hypertensive disease, referral from other hospital	1	Yes	Yes	Yes	1
4	Post-op elective total mandibulectomy	3	No	Yes	Yes	6
5	Peritonitis	Unknown	No	Yes	Yes	4
6	Post-op elective hemi-mandibulectomy	1	No	Yes	Yes	2
7	Eclampsia, ruptured uterus, acute renal failure, referral from other hospital	9	Yes	Yes	Yes	7
8	Poly-trauma, traffic accident, brain injury	17	No	Yes	Yes	48
9	Post-op elective thyroidectomy, huge non-toxic multi-nodular goitre	3	No	Yes	Yes	3
10	Post-op ameloblastoma	1	No	Yes	No	0
11	Post-op simple multi-nodular goitre	1	No	Yes	No	0
12	Poly-trauma, head injury, post-op explorative laparotomy	23 (7 during study month)	No	Yes	No	0
13	Post-op elective thyroidectomy, multi-nodular goitre	2	No	Yes	No	0
14	Uterine injury, post-op burst abdomen, re-laparotomy	Unknown	No	No	No	0
15	Intestinal obstruction, post laparotomy	Unknown	No	No	No	0

### Vital signs

**Figure 3:**
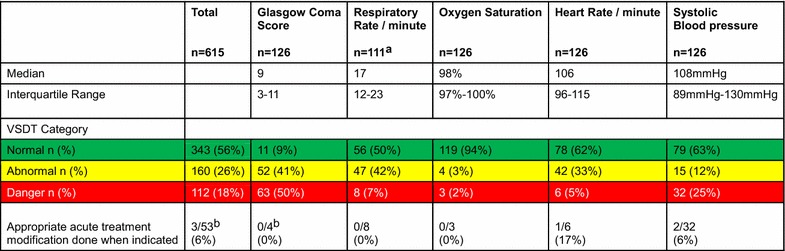
Distribution of vital signs. ^a^Respiratory rate was missing in 15 observation-timepoints. ^b^An acute treatment modification was indicated in 4 of the 63 observation-timepoints with GCS <9 as the patient had an unprotected airway. In the other 59 observation-timepoints acute treatment modification was not indicated according to VSDT. In these observation-timepoints 58 patients were intubated and one was in the lateral position.

In the 126 observation-timepoints there were data for 615 vital signs, of which 112 were severely deranged (18%) (Figure 3). In 53 of these severely deranged vital signs an acute treatment modification was indicated (9% of all observation-timepoints).

An appropriate treatment modification was done in 3 of these 53 occasions (6%).

A. For conscious level, data was complete. In 63 observation-timepoints a severely deranged vital sign was present (50%). In four of these cases the airway was unprotected. No acute treatment modification to protect the airway or any other action took place in these four cases.
B. Oxygen saturation data was complete and values were in the majority of cases normal. In 31% of cases saturation was 100%, and all of those patients were receiving oxygen therapy. Respiratory rate (RR) was available in 111 observation-timepoints. In none of the cases where severely deranged vital signs for RR (n = 8) or saturation (n = 3) were recorded was the oxygen delivery increased or the position of the patient changed. Nor were changes in ventilator settings made or any other action taken.
C. Heart rate and blood pressure data was complete. Severe hypotension was observed in 32 observation-timepoints (25%). In two cases 500 ml IV fluid was given. In the other 30 cases no acute treatment modification was made. One patient was already receiving low dose-Dopamine infusion, which was not increased when the patient became hypotensive. Six observations of severe tachycardia were found and in one case an appropriate acute treatment modification was made. In that case a bolus of 500 ml iv-fluid was given. In one case with tachycardia and anuria another action was taken; Pethidine was administered.

## Discussion

This observational study suggests that severely deranged vital signs occur frequently and rarely trigger acute treatment modifications on an ICU in a LIC. Of all documented vital signs 18% were severely deranged and for 9% an acute treatment modification was indicated. In only 6% of these situations was an appropriate acute treatment modification made.

To our knowledge, this is the first attempt to study the processes of care on an ICU in a LIC. We chose a vital-signs based methodology that provides information about illness severity and care processes that is realistic for the level of resources in healthcare in LICs. The results are striking and suggest an issue that has previously been neglected. The method may be used in larger clinical studies or in needs assessments of specific wards or ICUs.

The study is single centred and the sample size small, so generalising to other ICUs in Tanzania or other LICs is limited. Tracing medical records in hospitals in LICs is notoriously challenging, and we have missing data due to lost patient records and lost ICU observation charts. This missing data may be non-random, however, we believe it is unlikely that the patients with missing records would be much more stable or that their acute medical management would differ substantially from the patients in the study.

We used VSDT-danger signs to define severely deranged vital signs. Other methods could have been used such as MEWS [23], NEWS [24] or SATS [25]. We rejected these because they are more complicated compound scores and, while identifying critical illness, are not designed for translation into treatment suggestions.

We used VSDT-treatments to define the appropriate acute treatment modifications. The VSDT protocol is simple and standardised, and was developed to be practically suited as support for nurses on ICUs in LICs where doctors are scarce. Its simplicity could be problematic, as some deranged vital signs may warrant alternative treatments. We argue, however, that the initial treatment suggestions for airway threat, hypoxia and shock are uncontroversial and that the small minority of patients in which other initial treatment might be preferred would not be possible to identify without a much more advanced monitoring than is reasonable in this setting. Action would be better than no action for the vast majority of patients.

As many as 25% of observations indicated danger for hypoperfusion due to hypotension. In very few cases was appropriate action taken. If the indicated treatment modifications had been carried out, the subsequent increase in the amount of IV fluid boluses implies a potential to avoid organ damage [26]. Although the optimal volumes, types and monitoring methods of fluid therapy are debated, the use of IV fluids in non-cardiac causes of shock is established practice in HIC [27]. It is a cornerstone of the early goal directed therapy for sepsis [17] and in a sepsis bundle that showed markedly beneficial effects on mortality in Uganda [28]. It has recently been challenged in a paediatric population in LIC [29] but remains the global standard of care for adults [8, 30, 31].

Several authors have called for improved basic critical care in LICs [1, 6, 7, 32–34], which is likely to be a cost-effective part of health services [12, 35]. Multiple challenges to delivery of critical care in Tanzania have been identified, which may in part explain the results of this study. As well as the lack of financial and human resources at the national level [20], high lead times delaying treatment both pre- and in-hospital have been described [36]. A lack of routines and training for the identification and treatment of acute illness may be more important than availability of basic drugs and equipment [21, 37]. Fatalism and hierarchical structures may discourage action by the bedside staff [38, 39].

Ongoing initiatives are attempting to improve critical care in resource scarce settings. Guidelines have been written for sepsis management [8] and protocols for sepsis care have been trialled in Zambia [40] and Uganda [28]. A study of computer-programme-supported comprehensive critical care is underway that will also include hospitals in LICs [41].

We believe that improvement efforts should focus on simple, cheap interventions with a potential for marked gains in mortality and morbidity. Interventions that are realistic to implement and sustain in a setting where resources are scarce have a better chance of success. Our findings suggest that outcomes may be improved if severely deranged vital signs are identified and trigger acute treatment responses that can be carried out by the nurse bedside. A successful intervention addressing this need does not necessitate new treatments, diagnostic tools or staff and could be a cost-effective addition to the ordinary care.

## Conclusion

This study suggests that severely deranged vital signs are common and infrequently lead to acute treatment modifications in an ICU in a low-income country. A prospective interventional study with before and after-design studying the effect on mortality of the implementation of a vital signs directed protocol (VSDT) is underway. We believe that there is potential for the VSDT protocol to alter clinical practice. This may lead to improved outcomes and reduced mortality.

## Abbreviations

CT: Computed tomography
GCS: Glasgow Coma Scale
HIC: High-income country
ICU: Intensive Care Unit
LIC: low-income country
MEWS: Modified Early Warning Score
MKAIC: Muhimbili-Karolinska Anaesthesia and Intensive care Collaboration
MNH: Muhimbili National Hospital
NEWS: National Early Warning Score
RR: respiratory rate
SATS: South African Triage Scale
USD: United States Dollar
VSDT: vital signs directed therapy
