# Erratum to: Severely deranged vital signs as triggers for acute treatment modifications on an intensive care unit in a low-income country

**DOI:** 10.1186/s13104-017-2554-4

**Published:** 2017-08-02

**Authors:** Carl Otto Schell, Markus Castegren, Edwin Lugazia, Jonas Blixt, Moses Mulungu, David Konrad, Tim Baker

**Affiliations:** 1 Centre for Clinical Research Sörmland, Uppsala University, Uppsala, Sweden; 2 Department of Internal Medicine, Medicinkliniken, Nyköping Hospital, Sörmland County Council, 61185 Nyköping, Sweden; 3 Department of Anaesthesia and Intensive Care, Muhimbili University of Health and Allied Sciences, Dar es Salaam, Tanzania; 4 Department of Anaesthesia, Intensive Care and Surgical Services, Karolinska University Hospital, Stockholm, Sweden; 5 Department of Physiology and Pharmacology, Karolinska Institutet, Stockholm, Sweden; 6 Department of Anaesthesia and Intensive Care, Muhimbili National Hospital, Dar es Salaam, Tanzania; 7 Department of Physiology and Pharmacology, Karolinska Institutet, Stockholm, Sweden; 8 Global Health - Health Systems and Policy, Department of Public Health Sciences, Karolinska Institutet, Stockholm, Sweden

## Erratum to: BMC Res Notes (2015) 8:313 DOI 10.1186/s13104-015-1275-9

Following publication of the original article [1] a request was made to replace the image file for Fig. 1, which was found to be corrupt.

**Fig. 1:**
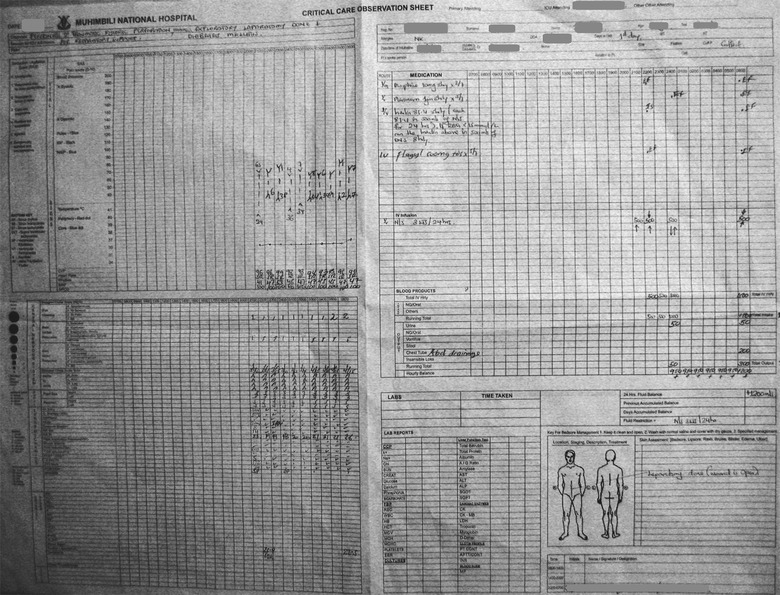
Data source: handwritten observation charts from the ICU. The ICU observation charts contain nurses’ records of vital signs, patients’ position, treatments administered and fluid balances every hour. There is also information on diagnosis, other medical treatments and care given

## References

[1] Schell CO, Castegren M, Lugazia E, Blixt J, Mulungu M, Konrad D, Baker T (2015). Severely deranged vital signs as triggers for acute treatment modifications on an intensive care unit in a low-income country. BMC Res Notes.

